# Comparative risk of severe constipation in patients treated with opioids for non-cancer pain: a retrospective cohort study in Northwest England

**DOI:** 10.1186/s12916-025-04118-7

**Published:** 2025-06-16

**Authors:** Belay Birlie Yimer, Mehreen Soomro, John McBeth, Carlos Raul Ramirez Medina, Mark Lunt, William G. Dixon, Meghna Jani

**Affiliations:** 1 Centre for Epidemiology Versus Arthritis, Centre for Musculoskeletal Research, The University of Manchester, Manchester, UK; 2 Department of Rheumatology, Salford Royal Hospital, Northern Care Alliance, Salford, UK; 3 NIHR Manchester Biomedical Research Centre, Manchester University NHS Foundation Trust, Manchester Academic Health Science Centre, Manchester, UK; 4 Division of Informatics, Imaging and Data Science, The University of Manchester, Manchester, UK; 5 Faculty of Medicine and Faculty of Engineering and Computer Science, The University of Southampton, Southampton, UK

**Keywords:** Opioids, Opiates, Constipation, Adverse events, Drug safety, Electronic health records, Morphine milligram equivalents, Opioid-related harms, Chronic pain

## Abstract

**Background:**

Constipation is a frequent adverse event associated with opioid medications that can have a considerable impact on patients’ quality of life. In patients who require opioids for pain relief, less is known about the risk conferred by specific opioids given their diverse pharmacology and the effect of daily dose and potency. The aim of the study was to evaluate the comparative risk of severe constipation by opioid type and dose in patients with non-cancer pain admitted to hospital.

**Methods:**

We conducted a retrospective cohort study using hospital electronic health records in Northwest England between December 1, 2009, and December 31, 2020. Patients who were ≥ 18 years and without a history of cancer were included. Opioid exposure was measured using administered drug information in hospital. The outcome was a severe constipation event defined as administration of an enema or suppository. Incidence rates by opioid use status, type of opioid class and morphine milligram equivalent (MME) per day were calculated, and a Cox regression model was used to determine associations with incident constipation after adjusting for confounders.

**Results:**

The study included 80,475 eligible patients who were administered an opioid in hospital. Compared to codeine, morphine (HR 1.59, 95% CI 1.45–1.74), oxycodone (HR 1.46, 95% CI 1.32–1.63), fentanyl (HR 1.37, 95% CI 1.14–1.64) and combination opioids (HR 1.85, 95% CI 1.66–2.06) were associated with a higher risk of constipation in the fully adjusted models. Tramadol demonstrated a significantly lower risk compared to codeine (HR 0.80, 95% CI 0.64–1.00). Higher opioid doses of more than ≥ 50 MME/day in comparison to < 50 MME/day were associated with an increased risk of constipation (compared to < 50 MME/day, 50 to < 120 MME/day: HR 1.95, 95% CI 1.78–2.15; ≥ 120 MME/day: HR 1.45, 95% CI 1.32–1.60).

**Conclusions:**

Morphine, oxycodone, fentanyl and combination opioids administration were associated with a significantly higher risk of severe constipation compared to codeine. Tramadol was associated with the lowest risk of the outcome compared to codeine. Patients on ≥ 50 MME/day experienced a higher risk of severe constipation compared to those on < 50 MME/day. These results can be used to guide better shared decisions with patients to balance benefit and harms of specific opioid types and doses.

**Supplementary Information:**

The online version contains supplementary material available at 10.1186/s12916-025-04118-7.

## Background

Opioids are frequently used in the management of pain internationally [1–3]. As a class of analgesic drugs, they are associated with a range of both serious adverse events that lead to hospitalisations and in some cases premature deaths [4, 5]. They may also be associated with non-life-threatening adverse events, which may be construed as less serious but can greatly impact patients’ quality of life. One such example is opioid-induced constipation, where the prevalence has been reported as high as 4 in 5 patients using self-reported data [6]. As well as the major impact on activities of daily living and patient-burden [7–9], opioid-induced constipation has also been associated with longer hospital stays, higher hospital costs and increased emergency department visits [10].

Opioid-induced constipation has been a challenging outcome for assessment in research due to considerable variations in how it has been defined or reported, the treatment setting, patient cohort and different opioid regimens with varied dosing. A systematic review of randomised controlled trials reported a prevalence of 30% in opioid-treated patients in older adults [11], whilst cross-sectional data from patient surveys to patients with chronic pain taking opioids reported a prevalence of between 70 and 81% [6, 12]. Estimates based solely on diagnostic codes within electronic health records (EHRs) are likely to under-represent the true prevalence both in primary and secondary health care records. This is because constipation may be deemed as less serious therefore not always coded in EHRs despite being mentioned in consultations, or patients may struggle openly discussing their bowel habits with their health care professional, thereby not attending clinic or not discussing it if they do attend. Whilst, as a class of analgesics, a high prevalence of constipation is reported amongst patients taking opioids, there are known differential pharmacological properties of each opioid that may impact the outcome. In patients who require an opioid for pain relief due to limited treatment options, information about the differential risk of different opioids can help tailor pain management to individual patient needs for better shared decisions before commencing these medications. This might include, for example, preferentially prescribing an opioid with a lower risk in someone who has a history of constipation.

Assessment of the comparative risk of adverse events in patients treated with opioids requires accurate information on both the exposure (opioids) and outcome (constipation). Whilst primary care EHRs and administrative records often provide information if the patient is electronically prescribed a medication or in some cases dispensation, whether the drug has been administered or not by the patient is usually absent. Hospital EHRs offers opportunities for measuring administered medication use thereby reducing exposure misclassification [13], especially when medications can be prescribed on an ‘as required’ basis [14]. Hospital EHRs also capture additional measures that may enable more precise methods for defining outcomes and the time of onset of the event.

The aim of this study was to evaluate the comparative risk of constipation in opioid-treated patients without prior cancer, who were administered an opioid in hospital. The specific objectives were to quantify this risk by opioid type and daily dose to enable more informed and tailored decisions between patients and health care professionals.

## Methods

### Study design and setting

We conducted a retrospective cohort study using secondary care EHRs data in a large tertiary care hospital in the Northwest of England between 1 December 2009 and 31 December 2020 (the study window). The hospital was an early adopter of electronic health records, and all medications throughout this period were prescribed electronically, including information recorded digitally on whether each drug was administered to the patient.

### Study population

Patients aged ≥ 18 years who were opioid users during hospital admissions within the study window were identified. Admissions that were least 1 day long, but not longer than 90 days were included. For each patient, only admission episodes with no prior malignancy ICD-10 code within 2 years of admission date were retained to establish a cohort of patients prescribed an opioid for non-cancer pain. These codes are provided in Additional file 1: Table S1. The date of initial opioid prescription was considered the index date for the corresponding patient. Follow-up time extended from the index date to the first outcome event of constipation, discharge date or end of the study (31st December 2020).

### Exposure

Administered opioid data was assessed at an individual level, including drug name, route of administration, dosage, and day and time of administration, from inpatient e-prescribing data. All opioids were extracted reflecting the frequently prescribed opioids used in the UK [1, 13]. Drugs used for opioid use disorder such as methadone or those used for anaesthetic induction in hospitals such as remifentanil were excluded. The e-prescribing data were processed and converted to daily opioid use data following the steps provided in Additional file 1: Fig. S1. Two measures to assess opioid exposure were considered. The first categorical opioid exposure measure categorised on-drug periods into monotherapy opioid medications (codeine, tramadol, morphine, fentanyl, buprenorphine and oxycodone), an ‘other’ opioids category (including diamorphine, dihydrocodeine, methanol, hydromorphone and pethidine) and a ‘combination opioid’ category (e.g. oxycodone and morphine or codeine and oromorph administered concomitantly). We performed a time-varying exposure analysis, meaning if patients switched to another opioid their risk attribution would change accordingly. To allow direct comparison of doses and opioid potencies across all opioids and formulations, we calculated morphine milligram equivalents (MME) for each prescription. The second measure was MME/day as defined as the daily dose for each prescription multiplied by the equivalent analgesic ratio as specified by the US Centres for Disease Control and Prevention (CDC) guidelines [15]. The MME dose was categorised as off-drug, < 50 (reference category), 50 to < 120 and ≥ 120 MME/day. The threshold of < 50 MME/day was used as a cut-off as it is the threshold above which the US CDC recommends caution as patients may be exposed to progressive increase in harms with no additional benefit in pain or function [15]. The Faculty of Pain Medicine in the UK currently suggests harms outweigh benefits when patients exceed 120 MME/day [16].

### Outcome

A patient was considered to have a constipation event if they had been administered an enema or a suppository drug (Additional file 1: Table S2). ICD-10 codes at discharge were not used to define constipation as the date corresponded to the date of coding following discharge, not the event date, which is required in a time to event analysis. By using this outcome definition, severe constipation was measured through administration (rather than simply prescription) of these drugs and captured a more accurate and time-sensitive representation of constipation events during the hospital stay.

### Covariates

Baseline characteristics, including age, sex, BMI, ethnicity and index of multiple deprivation (IMD), were measured and recorded on the hospital admission date. IMD, a relative measure of deprivation based on UK census data, was calculated using the first four digits of postcodes and reported in deciles. Conditions expected to be linked with constipation or to opioid administration, including irritable bowel syndrome, diabetes, hypothyroidism, multiple sclerosis, Parkinson’s disease, muscular dystrophy, Crohn’s disease, diverticulosis and chronic kidney disease, were defined using ICD-10 codes at discharge from the hospital within a 2-year window prior to the first admission of interest.

### Statistical analysis

The cohort’s baseline characteristics at the first index date were described using summary tables. In the analysis, we considered only the first admission episode. Crude incidence rates of constipation were calculated by opioid use status, type of opioid class and MME/day. To investigate the relationship between different types of opioid exposure and constipation, three separate Cox regression models were constructed. We performed a time-varying exposure analysis. The first model, referred to as ‘opioid exposed vs. not-exposed’, compared the risk of constipation amongst person time when any opioid was administered compared to person time when they were not exposed to opioids. The second model, the ‘opioid drug class model’, assessed the comparative risk of constipation associated with different opioid classes, using codeine as the referent group. Finally, the ‘opioid dose model’ examined the association between time varying levels of daily morphine milligram equivalents and the incidence of constipation. For each type of exposure measure, we fitted unadjusted and adjusted models (see Covariates section for the adjustment list and directed acyclic graph, Additional file 1: Fig. S2).

In all analyses, we considered a complete case analysis. In addition, all models used a risk attribution modelling approach whereby a patient was considered at risk of constipation for 1 day after the last day of opioid exposure to allow for the potentially long-lasting effects of these drugs. We performed an additional stratified analysis of patient who underwent major or orthopaedic surgery, as it could be an effect modifier in this study. All data analyses were performed using R (version 4.1.3).

## Results

### Study population and baseline characteristics

**Fig. 1:**
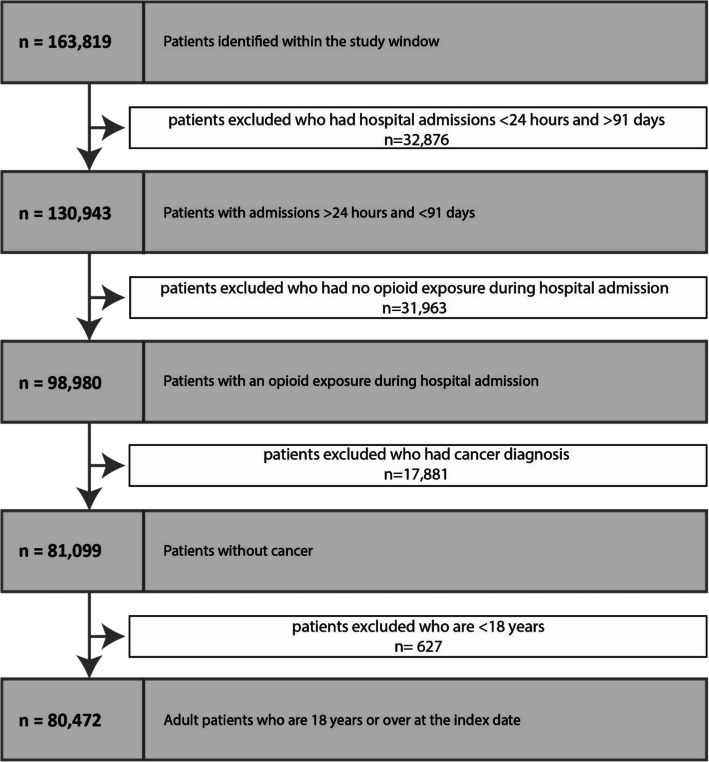
Cohort derivation diagram. Flow diagram demonstrating derivation of the final cohort

Within the study window, 80,475 patients fulfilled the inclusion criteria and were included in the analysis (Fig. 1). Baseline characteristics are described in Table 1. The median age was 54 years (standard deviation (SD), 20), with females representing 53% of the cohort. White was the most common ethnicity at 92%, followed by 3.7% Asian, 1.7% Black, 0.6% mixed and 1.1% other ethnic group. The predominant comorbidities recorded were musculoskeletal conditions (44%), diabetes type 1 or 2 (12%) and chronic kidney disease (5.4%). The highest proportion of the population were from the most deprived decile (rank = 1, 27%). The most commonly administered opioids used were codeine (37%) and morphine (30%), followed by oxycodone (11%). Patients on buprenorphine, oxycodone and fentanyl were older. The most common administration route of opioids was oral, except for buprenorphine (topical patch), morphine and fentanyl (intravenous as part of patient-controlled analgesia). The mean (SD) duration of opioid administration in days was 6.8 (10). Throughout the study period, 8% of patients were classified as having at least one episode of constipation. The baseline characteristics by MME/day at initiation are shown in Additional file 1: Table S3.

**Table 1:** Baseline characteristics of patients by opioid drug class at initiation

Characteristic	*N* = 80,475	Codeine, *N* = 29,855	Tramadol, *N* = 2575	Morphine, *N* = 24,283	Fentanyl, *N* = 3076	Buprenorphine, *N* = 505	Oxycodone, *N* = 8965	Other, *N* = 170	Combination, *N* = 11,040
Age, mean [SD] years	54 (20)	53 (20)	59 (18)	50 (18)	66 (17)	73 (18)	71 (16)	62 (16)	49 (19)
18–29	10,785 (13%)	4756 (16%)	182 (7.1%)	3600 (15%)	136 (4.4%)	NA	223 (2.5%)	NA	1871 (17%)
30–49	23,530 (29%)	8796 (29%)	603 (23%)	8738 (36%)	426 (14%)	68 (13%)	878 (9.8%)	32 (19%)	3987 (36%)
50–64	19,125 (24%)	6968 (23%)	719 (28%)	6712 (28%)	647 (21%)	53 (10%)	1390 (16%)	49 (29%)	2585 (23%)
65–79	16,733 (21%)	5943 (20%)	724 (28%)	3350 (14%)	1263 (41%)	136 (27%)	3440 (38%)	57 (34%)	1819 (16%)
> 80	10,302 (13%)	3392 (11%)	347 (13%)	1883 (7.8%)	604 (20%)	240 (48%)	3034 (34%)	24 (14%)	778 (7.0%)
Gender									
Female	42,657 (53%)	15,403 (52%)	1492 (58%)	12,528 (52%)	1734 (56%)	329 (65%)	5227 (58%)	98 (58%)	5846 (53%)
Male	37,812 (47%)	14,452 (48%)	1083 (42%)	11,755 (48%)	1342 (44%)	176 (35%)	3738 (42%)	72 (42%)	5194 (47%)
Ethnicity									
White	74,344 (92%)	27,192 (91%)	2417 (94%)	22,442 (92%)	2918 (95%)	483 (96%)	8500 (95%)	162 (95%)	10,225 (93%)
Asian	2961 (3.7%)	1234 (4.1%)	79 (3.1%)	882 (3.6%)	96 (3.1%)	11 (2.2%)	293 (3.3%)	NA	363 (3.3%)
Black	1387 (1.7%)	650 (2.2%)	34 (1.3%)	396 (1.6%)	28 (0.9%)	NA	74 (0.8%)	NA	198 (1.8%)
Mixed	497 (0.6%)	27,192 (91%)	16 (0.6%)	106 (0.4%)	15 (0.5%)	NA	29 (0.3%)	NA	53 (0.5%)
Other ethnic group	894 (1.1%)	242 (0.8%)	12 (0.5%)	149 (0.6%)	NA	NA	17 (0.2%)	NA	66 (0.6%)
Indices of multiple deprivations									
1 (most deprived)	21,568 (27%)	8396 (28%)	808 (31%)	6559 (27%)	635 (21%)	123 (24%)	2029 (23%)	58 (34%)	2958 (27%)
2	12,222 (15%)	4615 (15%)	375 (15%)	3732 (15%)	414 (13%)	81 (16%)	1308 (15%)	30 (18%)	1667 (15%)
3	9088 (11%)	3304 (11%)	275 (11%)	2794 (12%)	370 (12%)	68 (13%)	1002 (11%)	18 (11%)	1256 (11%)
4	8249 (10%)	3197 (11%)	246 (9.6%)	2462 (10%)	282 (9.2%)	37 (7.3%)	907 (10%)	16 (9.4%)	1101 (10.0%)
5	5660 (7.0%)	1964 (6.6%)	184 (7.1%)	1630 (6.7%)	269 (8.7%)	51 (10%)	767 (8.6%)	16 (9.4%)	779 (7.1%)
6	5222 (6.5%)	1862 (6.2%)	183 (7.1%)	1586 (6.5%)	211 (6.9%)	38 (7.5%)	590 (6.6%)	NA	744 (6.7%)
7	4750 (5.9%)	1625 (5.4%)	145 (5.6%)	1439 (5.9%)	226 (7.3%)	19 (3.8%)	629 (7.0%)	NA	663 (6.0%)
8	5180 (6.4%)	1787 (6.0%)	167 (6.5%)	1526 (6.3%)	247 (8.0%)	46 (9.1%)	706 (7.9%)	NA	695 (6.3%)
9	4301 (5.3%)	1545 (5.2%)	111 (4.3%)	1284 (5.3%)	199 (6.5%)	24 (4.8%)	553 (6.2%)	NA	579 (5.2%)
10	3435 (4.3%)	1296 (4.3%)	64 (2.5%)	984 (4.1%)	183 (5.9%)	14 (2.8%)	421 (4.7%)	NA	466 (4.2%)
Comorbidities									
Diabetes (type I or 2)	9557 (12%)	3277 (11%)	450 (17%)	2410 (9.9%)	517 (17%)	108 (21%)	1784 (20%)	35 (21%)	976 (8.8%)
Chronic kidney disease	4347 (5.4%)	1216 (4.1%)	164 (6.4%)	653 (2.7%)	332 (11%)	78 (15%)	1539 (17%)	17 (10%)	348 (3.2%)
Musculoskeletal (MSK) conditions	35,105 (44%)	11,147 (37%)	1542 (60%)	10,077 (41%)	1827 (59%)	292 (58%)	5192 (58%)	82 (48%)	4944 (45%)
Inflammatory bowel syndrome	1192 (1.5%)	372 (1.2%)	60 (2.3%)	408 (1.7%)	48 (1.6%)	11 (2.2%)	114 (1.3%)	4 (2.4%)	175 (1.6%)
Hypothyroidism	3795 (4.7%)	1307 (4.4%)	161 (6.3%)	937 (3.9%)	188 (6.1%)	51 (10%)	677 (7.6%)	16 (9.4%)	457 (4.1%)
Multiple sclerosis	382 (0.5%)	162 (0.5%)	35 (1.4%)	98 (0.4%)	11 (0.4%)	–	36 (0.4%)	–	37 (0.3%)
Parkinson’s disease	654 (0.8%)	220 (0.7%)	23 (0.9%)	159 (0.7%)	37 (1.2%)	15 (3.0%)	142 (1.6%)	–	57 (0.5%)
Crohn’s disease	1030 (1.3%)	303 (1.0%)	49 (1.9%)	385 (1.6%)	59 (1.9%)	–	86 (1.0%)	–	138 (1.3%)
Muscular dystrophy	80 (< 0.1%)	26 (< 0.1%)	–	23 (< 0.1%)	–	–	15 (0.2%)	–	–
Diverticulitis	995 (1.2%)	327 (1.1%)	51 (2.0%)	234 (1.0%)	41 (1.3%)	20 (4.0%)	215 (2.4%)	–	106 (1.0%)
Major or orthopaedic Surgery during admission	16,414 (20%)	4258 (14%)	367 (14%)	6336 (26%)	1011 (33%)	15 (3.0%)	1522 (17%)	9 (5.3%)	2896 (26%)
Hospitalisation length, mean [SD] days	8 (12)	7 (12)	8 (12)	8 (12)	11 (14)	14 (17)	13 (16)	9 (10)	7 (10)
Most common route	Oral	Oral	Oral	Intravenous/injection	Intravenous/injection	Topical (patch)	Oral	Oral	Oral
Morphine milligram equivalent (MME)/day at initiation									
< 50	68,840 (85.5%)	29,851 (100%)	2570 (99.8%)	17,983 (74.1%)	915 (29.7%)	455 (90.1%)	8467 (94.4%)	165 (97.1%)	8429 (76.3%)
50 to < 120	4946 (6.1%)	3 (0%)	5 (0.2%)	2239 (9.2%)	544 (17.7%)	8 (1.6%)	406 (4.5%)	3 (1.8%)	1738 (15.7%)
≥ 120	6689 (8.3%)	1 (0%)	0 (0.0%)	4061 (16.7%)	1617 (52.6%)	42 (8.3%)	92 (1.0%)	2 (1.2%)	873 (7.9%)

*Abbreviations*: *NA*, not available. These data are not available due to small cells/statistical disclosure control to protect patient confidentiality. The socioeconomic deprivation index measure by IMD was largely complete, with only 1.0% of data missing

### Comparative risk of constipation associated with different opioids

The results for the association between the comparative risk of constipation with various opioids are presented in Table 2. In the ‘opioid exposed vs. not-exposed’ analysis, the crude rates of constipation in exposed and non-exposed person time were 12.0 events/1000 person-days and 6.44 events/1000 person-days, respectively. The adjusted hazard ratio (HR) was 1.75 (95% confidence interval [CI], 1.62 to 1.90) in the opioid-exposed group compared to unexposed.

**Table 2:**
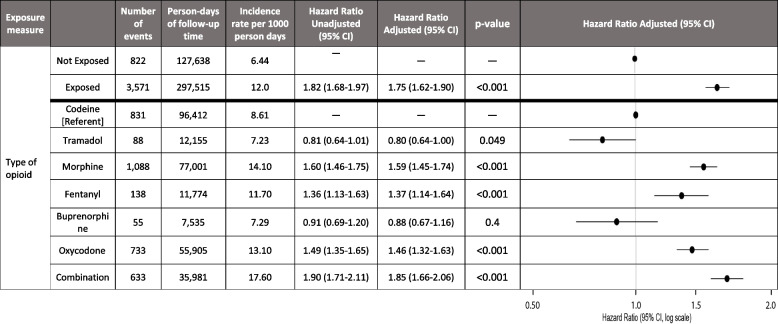
Association between administered opioid exposure and severe constipation

Other opioids (diamorphine, dihydrocodeine, methanol, hydromorphone, pethidine) are excluded from this table due to low count

In the adjusted opioid drug class analysis, the most commonly administered drug, codeine, had a crude incidence rate of 8.61 events/1000 person-days, morphine 14.1 events/1000 person-days, oxycodone 13.1 events/1000 person-days, fentanyl 11.7 events/1000 person-days, combination opioid therapy 17.6 events/1000 person-days and tramadol 7.2/1000 person-days. Patients administered fentanyl, morphine, oxycodone and combination opioids had a significantly higher risk of experiencing constipation compared to those on codeine. Adjusted hazard ratios for specific drugs included fentanyl (HR 1.37, 95% CI, 1.14 to 1.64), morphine (HR 1.59, 95% CI, 1.45 to 1.74), oxycodone (HR 1.46, 95% CI, 1.32 to 1.63) and combination opioid therapy (HR 1.85, 95% CI, 1.66 to 2.06). There was a significantly lower risk of constipation associated with tramadol compared to codeine (HR, 0.80, 95% CI, 0.64 to 1.00) (Table 2). In the sensitivity analysis restricting to only patients undergoing major and orthopaedic surgery, the results and direction of risk remain unchanged with wider confidence intervals due to fewer events (Additional file 1: Table S4). In these patients, there continued to be a significantly lower risk of constipation with tramadol compared to codeine (HR 0.46, 95% CI 0.23–0.95).

In the adjusted MME analysis, patients taking < 50 MME/day had a crude incidence rate of 9.96 events/1000 person-days compared to those taking 50 to < 120 MME (21.9 events/1000 person-days) and ≥ 120 MME/day (15.6 events/1000 person-days). There was a significantly higher risk of constipation in patients administering higher doses ≥ 50 MME/day compared to those taking lower doses (< 50 MME/day) (Table 3). Compared to < 50 MME/day, the adjusted HR for 50 to < 120 MME/day was 1.95 (95% CI, 1.78 to 2.15), and for > 120 MME/day 1.45 (95% CI, 1.32 to 1.60) (Table 3). Adjusted hazard ratios with covariate point estimates for drug type and MME/day are presented in Additional file 1: Tables S5 and S6, respectively.

**Table 3:**
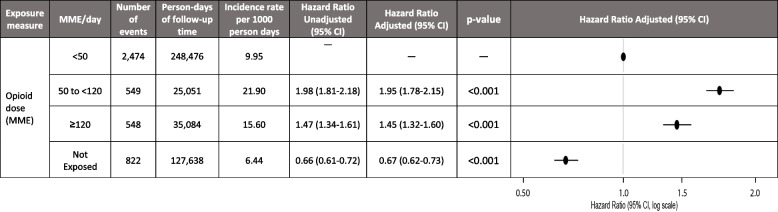
Association between morphine milligram equivalents per day thresholds and severe constipation

## Discussion

In this retrospective cohort study, the administration of specific opioid drugs and regimens were associated with a differential risk of severe constipation. Patients who were administered morphine, oxycodone, fentanyl or a combination of opioids had a higher risk of constipation compared to patients administered codeine after adjusting for confounding factors. Tramadol administration was associated with a lower risk of severe constipation compared to codeine. Additionally, patients administering opioids of more than 50 MME per day were at higher risk of developing severe constipation than those who did not.

Opioids, as a class of medications, are well known to be associated with constipation and other less common gastrointestinal effects, as they bind not only to mu-opioid receptors present in the brain and spinal cord but also in the gut. This results in reduced peristalsis (gastric motility) and increases fluid absorption in the gastrointestinal tract, leading to hard stools that are difficult to pass [8]. However, the extent to which they activate these receptors in the gastrointestinal tract can vary depending on the pharmacological action of the specific drug. The differences in pharmacological profiles between opioids can, therefore, lead to differential risks, as reported in our study. Tramadol has a different mechanism of action to other opioids as, alongside its opioid receptor activity, it is also a partial inhibitor of serotonin and norepinephrine reuptake [17]. This may explain why it was associated with a significantly lower risk of severe constipation compared to codeine in this study. Buprenorphine is a partial agonist at the mu-opioid receptor and an antagonist at the kappa-opioid receptor [18], which may be associated with the lower incidence rate of events compared to codeine and the statistically non-significant lower adjusted HR. Often considered a less potent opioid [19], codeine was not associated with the lowest risk of this outcome.

Previous work assessing the comparative risk of constipation between different opioids has been conflicting. This is in part due to the diagnosis of opioid-induced constipation in the literature being complicated by the lack of a consensus definition and underreporting when reliant on diagnostic codes within EHRs. A previous study comparing the risk of codeine vs. tramadol using primary care EHRs from Catalonia in Spain reported no significant differences in risk of constipation between drugs [20]. However, the outcome definition of constipation in this study was based on ICD-10 codes, which is likely to be an under representation of those who have this specific outcome due to the reasons discussed above. For instance, the incidence rate of any constipation event in this study for codeine using ICD-10 codes was 6.41 per 1000 person-years [20]; in our study, the rate for severe constipation on codeine was 8.61 per 1000 person-days which is considerably higher and more clinically plausible in keeping with patient self-reports, suggesting underreporting of this outcome using health codes alone. Another study using administrative health records in 14,491 patients with osteoarthritis found rates of specific opioid-associated outcomes that included constipation, nausea and others were lower in patients initiated on tramadol compared to those initiated on all other opioids [21], which is more consistent with our work. Neither study however was able to account for the effect of daily dose on the outcome.

Higher MME/day can be associated with a range of adverse events, with recommended MME/day thresholds advising caution and careful monitoring varying between 50 and 120 MME/day internationally [1, 15, 16]. Our study demonstrated that the risk of severe constipation associated with opioids increased significantly in patients on ≥ 50 MME/day underscoring the importance of opioid stewardship [22], based on the individual patient. Patients on higher opioid doses may require vigilant monitoring and proactive management for constipation to mitigate these symptoms and prevent potential complications. Several studies reporting on opioid-associated adverse events do not report on the effect of MME/day, as it either be especially challenging to prepare such data with a combination of ‘as required’ and regular medications that may overlap [14] or due to considerable missing information within the prescribing/dispensing data. Indeed, a recent Cochrane review evaluating the efficacy and safety of high-dose opioids in chronic non-cancer pain concluded that whilst such patients are commonly on high-dose opioids, studies rarely reported on dose [23]. Therefore, it was unable to draw definitive conclusions based on high-quality scientific evidence.

The major strengths of this study include the use of administered drug data rather than prescribing data, which provided more accurate exposure information and reduced the risk of exposure misclassification, especially for an ‘as required’ medication. Additionally, we used a novel approach in defining the outcome of constipation based on administered suppository and enema use, which is less reliant on health codes or the patient’s willingness to approach their health care professional. We also incorporated the effects of dose and potency, by evaluating the effect of MME/day on the outcome using clinically meaningful thresholds. Previous work evaluating the effect of opioid type on adverse events such as constipation has used crude proxy measures for assessing dose instead such as dispensed packages of opioids [20]. The findings of this work need to be interpreted in the context of its limitations. We were not able to stratify if the patient had previously been exposed to an opioid pre-admission to hospital or had a previous history of constipation. Whilst our choice of outcome had specific advantages, it meant that only severe constipation episodes would be captured if a suppository or enema was administered. Mild to moderate constipation that may respond to a laxative was not assessed. Severe constipation was defined as per administered medication data from EHRs rather than fulfilling diagnostic criteria. Whilst there are diagnostic criteria for different types of constipation that exist such as the Rome-IV diagnostic criteria, they are based on information about the patient straining, sensations of anorectal blockage/incomplete evacuation and measurement of stools against the Bristol stool index. As study used routinely collected data from EHRs which not include all the data items needed to apply the Rome-IV criteria, it was not possible to apply such criteria [24]. Including laxatives for defining the outcome was considered and rejected, however, as they are often prescribed and administered in patients on opioids as prophylaxis rather than treatment [25]. Effect modification through concomitant use of specific medications that may influence the outcome, such as anticholinergics or time varying laxative use at the time of the opioid use, was not assessed. As the length of stay in hospital for most patients was fairly modest, the effect of long-term opioid use on the outcome (e.g. 3 months or over [26]) was out of scope of this study. The specific opioids evaluated in this study reflect the opioids most frequently prescribed in the UK, which include codeine, tramadol and morphine [1]. Whilst our previous work has shown that there are clear overlaps between the first-line opioid prescription preferences in the UK, Canada, the USA and Taiwan [2, 27], the frequency of opioid type can vary across jurisdictions. Given there are known pharmacological differences between opioid types [28], these data cannot be extrapolated to drugs such as hydrocodone and hydromorphone which are not commonly prescribed in the UK but more frequently prescribed in North America [2, 27]. Patients undergoing specific types of surgery such as bowel resections may have a different baseline risk of constipation depending on whether this was a symptom before administration of opioids. However, the most common types of major/orthopaedic surgery from a previous study using the same data source describing post-operative use of opioids were spinal decompressions, neurosurgery, knee/hip replacement and cholecystectomy reflecting the surgical expertise in this centre [13]. Finally, lifestyle factors such as mobility and diet are known to impact the risk of constipation and are not measured in EHRs; therefore, their impact could not be examined.

The wider implications of our study are important for both clinical practice and public health. By identifying the comparative risks of constipation amongst different opioid medications and doses, healthcare professionals can better tailor pain management strategies to individual needs, potentially minimising the occurrence of this common adverse effect that can impact patient lives. Additionally, our study suggests the merit of considering tramadol as a lower-risk alternative in those requiring opioids, which may be especially valuable for patients already at risk for or concerned about this side effect. Furthermore, the dose–response relationship identified calls for prescribers to be cautious with opioid dosing, aiming for the lowest effective dose to manage pain whilst mitigating the risk of constipation. The UK Faculty of Pain Medicine treatment recommendations currently have a dose threshold of 120 MME/day as the limit above which harms outweigh benefits and opioid tapering should be considered [16]. Our study is more aligned with a lower recommended threshold of < 50 MME/day, consistent with the US Centres for Disease Control and Prevention opioid prescribing guidelines for pain [15]. Increased awareness and proactive management of constipation as a side effect of opioid therapy can improve patient outcomes and adherence to pain management regimens.

## Conclusions

Our study, to our knowledge, is the largest study evaluating the comparative safety of constipation across different opioids in patients with non-cancer pain. We used hospital EHRs to define exposure using administrations of different opioids to reduce exposure misclassification and the administration of suppositories to measure the outcome of severe constipation accurately at the time of the event. Compared to codeine, administration of tramadol conferred a lower risk of severe constipation whilst morphine, oxycodone, fentanyl and combination of opioids were associated with a higher risk. Additionally, a daily MME of ≥ 50 per day was also associated with a higher risk of severe constipation compared to those on daily doses of < 50 MME/day. The results can help more tailored prescribing based on individual patient needs and allow more informed shared decisions that can prompt discussions about opioid tapering where appropriate.

## Supplementary Information

Additional file 1: Fig. S1. The data preparation steps for processing and converting electronic administration data into daily opioid dose. Table S1 ICD-10 codes for malignancy. Table S2 Suppositories used to define the outcome of severe constipation. Fig. S2 Directed acyclic graph demonstrating potential effect of confounders. Table S3 Baseline characteristics of study patients by morphine milligram equivalents. Table S4 Association between administered opioid exposure and constipation in patients with major or orthopaedic surgery during admission. Table S5 Adjusted hazard ratios for severe constipation risk amongst patients exposed to different opioids (including covariate results). Table S6 Association between morphine milligram equivalents per day thresholds and severe constipation (including covariates).

## Data Availability

The data that support the findings of this study are available from the Northern Care Alliance but restrictions apply to the availability of these data, which were used under license for the current study and so are not publicly available. Data are however available from the authors upon reasonable request and with permission of the Northern Care Alliance (https://www.northerncarealliance.nhs.uk/contact-us). The data for the study were accessed and analysed within a Secure Research Environment that is hosted by Northern Care Alliance. In order to access the data, researchers will need to be Office of National Statistics Safe Researcher accredited (https://www.ons.gov.uk/aboutus/whatwedo/statistics/requestingstatistics/secureresearchservice/becomeanaccreditedresearcher) and need to have undergone appropriate background checks prior to permission being granted from the host organisation.

## Abbreviations

CI: Confidence interval
HR: Hazard ratio
IMD: Index of multiple deprivation
EHR: Electronic health records
MME: Morphine milligram equivalents
NA: Not available
SD: Standard deviation
