# Supplementary material for: Comparative risk of severe constipation in patients treated with opioids for non-cancer pain: a retrospective cohort study in Northwest England
Source: BMC Med. 2025 Jun 16;23:288. doi: 10.1186/s12916-025-04118-7 (PMC12168406; doi:10.1186/s12916-025-04118-7)
Supplement: Supplementary file 1 — Additional file 1: Fig. S1. The data preparation steps for processing and converting electronic administration data into daily opioid dose. Table S1 ICD-10 codes for malignancy. Table S2 Suppositories used to define the outcome of severe constipation. Fig. S2 Directed acyclic graph demonstrating potential effect of confounders. Table S3 Baseline characteristics of study patients by morphine milligram equivalents. Table S4 Association between administered opioid exposure and constipation in patients with major or orthopaedic surgery during admission. Table S5 Adjusted hazard ratios for severe constipation risk amongst patients exposed to different opioids (including covariate results). Table S6 Association between morphine milligram equivalents per day thresholds and severe constipation (including covariates). [file 12916_2025_4118_MOESM1_ESM.docx]

# Supplementary Material

**Supplementary Figure 1: The data preparation steps for processing and converting electronic administration data into daily opioid dose**


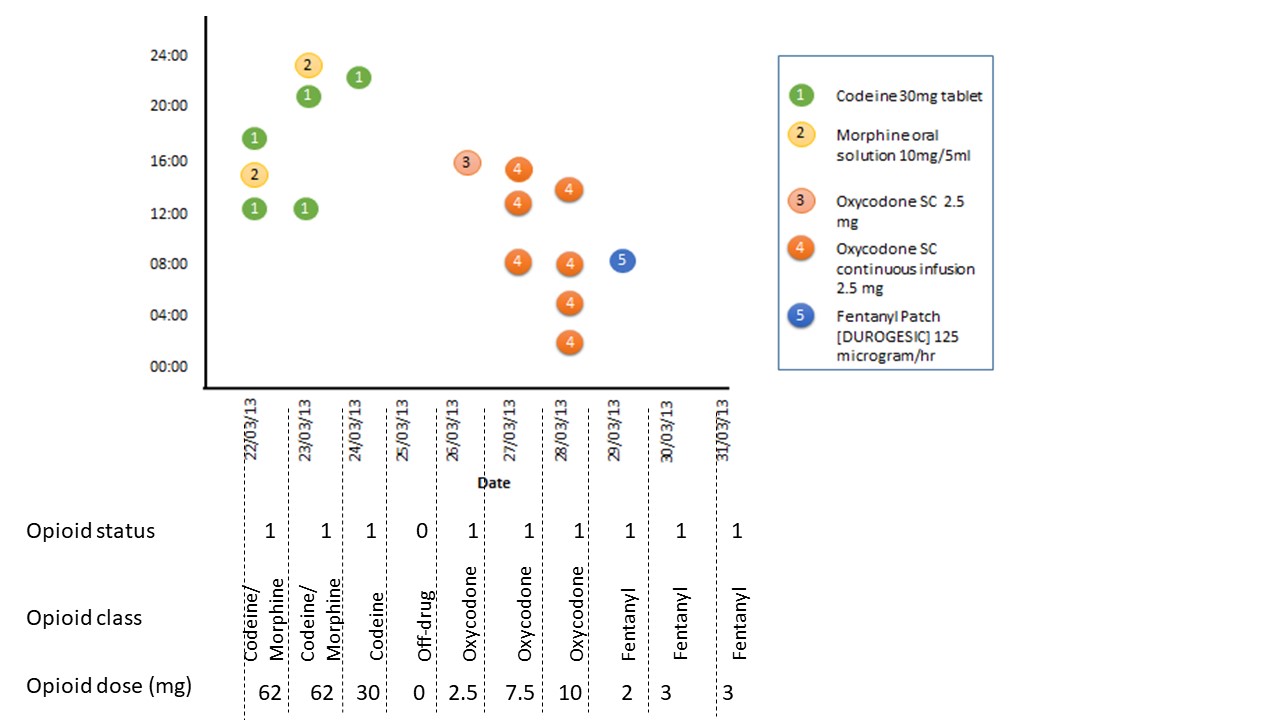


This figure illustrates an example of opioid administration between March 22 and March 31, 2013, with the y-axis representing time and the x-axis representing the dates. The coloured numbered circles indicate different opioids: (1) Codeine 30 mg (green), (2) Morphine oral solution 10 mg/5 ml (yellow), (3) Oxycodone SC 2.5 mg (light orange), (4) Oxycodone SC continuous infusion 2.5 mg (dark orange), and (5) Fentanyl Patch 125 mcg/hr (blue). The table below details opioid status (on/off), opioid class, and daily dose (mg), showing dose adjustments and medication switches. Initially, the patient received codeine and morphine, followed by a brief opioid-free period, before transitioning to oxycodone and then fentanyl, reflecting switches in pain management treatment and the importance of a time-varying exposure analysis.

**Supplementary Table 1 ICD-10 codes for malignancy**

| **ICD-10 codes** |
| --- |
| C00 to C26 |
| C30 to C41 |
| C43 to C58 |
| C60 to C96 |
| C7A |
| C7B |
| D00 to D49 |
| D3A |

**Supplementary Table 2: Suppositories used to define the outcome of severe constipation**

| **Type of medication** | **Information about formulations/ trade names** |
| --- | --- |
| Glycerol suppositories | <https://www.medicines.org.uk/emc/product/3535/smpc> |
| Micralax micro-enema | <https://www.medicines.org.uk/emc/product/9099/smpc> |
| Bisacodyl suppositories | <https://www.medicines.org.uk/emc/product/5505/smpc> |
| Phosphate Enema | <https://www.medicines.org.uk/emc/product/3772>) |
| Micolette Micro-enema | <https://www.medicines.org.uk/emc/product/9099/smpc> |

**Supplementary Figure 2: Directed Acyclic Graph demonstrating potential effect of confounders**


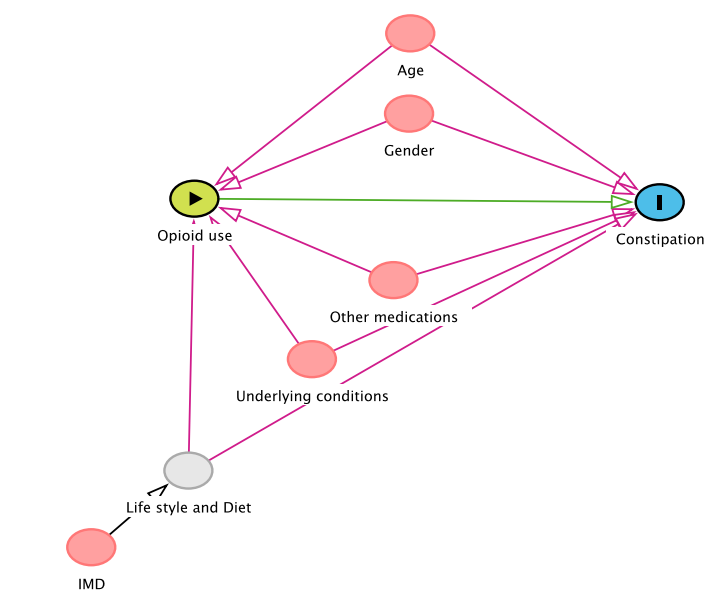


**Supplementary Table 3: Baseline characteristics of Study Patients by Morphine Milligram Equivalents**

| **Characteristic** | **120+ MME/day**  **n= 6,688** | **50 to < 120 MME/day**  **n = 4,946** | **<50 MME/day**  **n = 68,835** |
| --- | --- | --- | --- |
| Age, mean [SD] | 51 (16) | 49 (17) | 55 (20) |
| 18-29 | 789 (12%) | 714 (14%) | 9,281 (13%) |
| 30-49 | 2,272 (34%) | 1,966 (40%) | 19,290 (28%) |
| 50-64 | 2,079 (31%) | 1,249 (25%) | 15,795 (23%) |
| 65-79 | 1,338 (20%) | 823 (17%) | 14,571 (21%) |
| >80 | 210 (3.1%) | 194 (3.9%) | 9,898 (14%) |
| **Gender** |  |  |  |
| Female | 3,539 (53%) | 2,536 (51%) | 36,582 (53%) |
| Male | 3,149 (47%) | 2,410 (49%) | 32,253 (47%) |
| **Ethnicity** |  |  |  |
| White | 6,298 (94%) | 4,683 (95%) | 63,358 (92%) |
| Asian | 208 (3.1%) | 126 (2.5%) | 2,627 (3.8%) |
| Black | 82 (1.2%) | 47 (1.0%) | 1,257 (1.8%) |
| Other Ethnic groups | 43 (0.6%) | 46 (0.9%) | 805 (1.2%) |
| Mixed | 37 (0.6%) | 25 (0.5%) | 435 (0.6%) |
| **Indices of multiple deprivations** |  |  |  |
| 1 (Most deprived) | 1,679 (25%) | 1,293 (26%) | 18,594 (27%) |
| 2 | 966 (14%) | 722 (15%) | 10,534 (15%) |
| 3 | 806 (12%) | 579 (12%) | 7,702 (11%) |
| 4 | 574 (8.6%) | 465 (9.4%) | 7,209 (10%) |
| 5 | 492 (7.4%) | 356 (7.2%) | 4,812 (7.0%) |
| 6 | 415 (6.2%) | 331 (6.7%) | 4,475 (6.5%) |
| 7 | 478 (7.1%) | 333 (6.7%) | 3,938 (5.7%) |
| 8 | 448 (6.7%) | 330 (6.7%) | 4,402 (6.4%) |
| 9 | 387 (5.8%) | 263 (5.3%) | 3,651 (5.3%) |
| 10 | 315 (4.7%) | 213 (4.3%) | 2,907 (4.2%) |
| **Comorbidities** |  |  |  |
| Diabetes (Type I or 2) | 734 (11%) | 464 (9.4%) | 8,359 (12%) |
| Chronic Kidney Disease | 141 (2.1%) | 124 (2.5%) | 4,082 (5.9%) |
| Musculoskeletal conditions | 3,737 (56%) | 2,430 (49%) | 28,936 (42%) |
| Inflammatory Bowel Syndrome | 90 (1.3%) | 90 (1.8%) | 1,012 (1.5%) |
| Hypothyroidism | 247 (3.7%) | 182 (3.7%) | 3,365 (4.9%) |
| Multiple Sclerosis | 21 (0.3%) | 23 (0.5%) | 338 (0.5%) |
| Parkinson’s Disease | 32 (0.5%) | 12 (0.2%) | 610 (0.9%) |
| Crohn’s Disease | 165 (2.5%) | 70 (1.4%) | 795 (1.2%) |
| Muscular Dystrophy | 10 (0.1%) | <10 | 67 (<0.1%) |
| Diverticulitis | 46 (0.7%) | 47 (1.0%) | 902 (1.3%) |

**Supplementary Table 4: Association between administered opioid exposure & constipation in patients with major or orthopaedic surgery during admission**

| **Drug exposure** | **Number of events** | **Person-days of follow-up time** | **Incidence rate per 1000 person days** | **Hazard Ratio Unadjusted (95% CI)** | **Hazard Ratio Adjusted (95% CI)** | **p-value** |
| --- | --- | --- | --- | --- | --- | --- |
| Codeine [Referent] | 129 | 10,888 | 11.85 | — | — | — |
| Tramadol | 8 | 1,275 | 6.27 | 0.46 (0.23-0.95) | 0.45 (0.22, 0.92) | 0.029 |
| Morphine | 314 | 20,005 | 15.70 | 1.21 (0.99-1.49) | 1.16 (0.94-1.43) | 0.2 |
| Fentanyl | 43 | 2,465 | 17.44 | 1.59 (1.11-2.26) | 1.61 (1.12-2.31) | 0.010 |
| Buprenorphine | 1 | 554 | 1.80 | 0.17 (0.02-1.24) | 0.17 (0.02- 1.28) | 0.086 |
| oxycodone | 168 | 10,629 | 15.81 | 1.13 (0.89-1.42) | 1.17 (0.92-1.50) | 0.2 |
| Combination | 196 | 8,768 | 22.35 | 1.55 (1.24-1.95) | 1.49 (1.19-1.87) | <0.001 |

Other opioids (diamorphine, dihydrocodeine, methanol, hydromorphone, pethidine) are excluded from this table due to low count.

**Supplementary Table 5: Adjusted hazard ratios for severe constipation risk among patients exposed to different opioids (including covariate results)**

| Characteristic | Hazard Ratio Adjusted (95% CI) | p-value |
| --- | --- | --- |
| Opioid Type | | |
| Codeine [Referent] | — |  |
| Tramadol | 0.8 (0.6, 1.0) | 0.05 |
| Morphine | 1.6 (1.4, 1.7) | <0.001 |
| Fentanyl | 1.3 (1.1, 1.6) | <0.001 |
| Buprenorphine | 0.8 (0.6, 1.1) | 0.3 |
| Oxycodone | 1.4 (1.3, 1.6) | <0.001 |
| Combination | 1.8 (1.6, 2.0) | <0.001 |
| Age | | |
| 18-29 [Referent] | — | — |
| 30-49 | 0.9 (0.82, 1.03) | 0.1 |
| 50-64 | 0.9 (0.81, 1.03) | 0.1 |
| 65-79 | 0.9 (0.83, 1.06) | 0.3 |
| >80 | 0.9 (0.85, 1.11) | 0.7 |
| Gender | | |
| Female [Referent] | — |  |
| Male | 0.9 (0.84, 0.95) | <0.001 |
| Ethnicity | | |
| White [Referent] | - | - |
| Asian | 1.3 (1.13, 1.52) | <0.001 |
| Black | 1.2 (0.99, 1.56) | 0.062 |
| Mixed | 0.9 (0.62, 1.52) | >0.9 |
| NA | 0.9 (0.57, 1.57) | 0.8 |
| Other Ethnic group | 1.2 (0.93, 1.67) | 0.14 |
| Index of Multiple Deprivation Decile | | |
| 1 [Referent] | — |  |
| 2 | 1.1 (1.02, 1.25) | 0.017 |
| 3 | 1.1 (1.01, 1.25) | 0.035 |
| 4 | 1.2 (1.06, 1.33) | 0.002 |
| 5 | 1.3 (1.11, 1.42) | <0.001 |
| 6 | 1.2 (1.07, 1.40) | 0.004 |
| 7 | 1.4 (1.24, 1.61) | <0.001 |
| 8 | 1.3 (1.12, 1.45) | <0.001 |
| 9 | 1.4 (1.25, 1.64) | <0.001 |
| 10 | 1.5 (1.34, 1.78) | <0.001 |
| Comorbidities | | |
| Diabetes | 0.9 (0.77, 0.93) | <0.001 |
| CKD | 0.8 (0.68, 0.87) | <0.001 |
| MSK | 1.2 (1.11, 1.26) | <0.001 |
| IBS | 1.1 (0.87, 1.40) | 0.4 |
| Hypothyroidism | 0.9 (0.86, 1.12) | 0.8 |
| Multiple Sclerosis | 1.2 (0.88, 1.78) | 0.2 |
| Parkinson's Disease | 1.2 (0.92, 1.52) | 0.2 |
| Crohn's Disease | 0.5 (0.39, 0.69) | <0.001 |
| Muscular Dystrophy | 0.4 (0.09, 1.47) | 0.2 |
| Diverticulosis | 0.9 (0.67, 1.12) | 0.3 |

**Supplementary Table 6: Association between Morphine Milligram Equivalents per day thresholds & severe constipation (including covariates)**

| **Characteristic** | **Hazard Ratio Adjusted (95% CI)** | **p-value** |
| --- | --- | --- |
| MME | | |
| [0 - 50) | — |  |
| [50 - 120) | 1.9 (1.78, 2.15) | <0.001 |
| 120+ | 1.4 (1.32, 1.60) | <0.001 |
| Not Exposed | 0.6 (0.62, 0.73) | <0.001 |
| Age | | |
| 18-29 [Referent] | — | — |
| 30-49 | 0.9 (0.81, 1.01) | 0.087 |
| 50-64 | 0.9 (0.80, 1.01) | 0.076 |
| 65-79 | 0.9 (0.80, 1.02) | 0.11 |
| >80 | 0.9 (0.84, 1.09) | 0.5 |
| Gender | | |
| Female | — |  |
| Male | 0.9 (0.84, 0.95) | <0.001 |
| Ethnicity | | |
| White [Referent] | — |  |
| Asian | 1.3 (1.14, 1.53) | <0.001 |
| Black | 1.3 (1.00, 1.58) | 0.05 |
| Mixed | 0.9 (0.62, 1.49) | 0.9 |
| NA | 0.9 (0.55, 1.52) | 0.7 |
| Other Ethnic group | 1.2 (0.95, 1.70) | 0.11 |
| Index of Multiple Deprivation Decile | | |
| 1 [Referent] | — |  |
| 2 | 1.1 (1.03, 1.25) | 0.014 |
| 3 | 1.1 (1.01, 1.26) | 0.029 |
| 4 | 1.2 (1.07, 1.34) | 0.001 |
| 5 | 1.3 (1.12, 1.42) | <0.001 |
| 6 | 1.2 (1.06, 1.39) | 0.005 |
| 7 | 1.4 (1.26, 1.63) | <0.001 |
| 8 | 1.3 (1.13, 1.46) | <0.001 |
| 9 | 1.4 (1.26, 1.65) | <0.001 |
| 10 | 1.6 (1.36, 1.81) | <0.001 |
| Comorbidities |  |  |
| Diabetes | 0.9 (0.78, 0.94) | <0.001 |
| CKD | 0.8 (0.70, 0.89) | <0.001 |
| MSK | 1.2 (1.12, 1.27) | <0.001 |
| IBS | 1.1 (0.88, 1.41) | 0.4 |
| Hypothyroidism | 1.0 (0.87, 1.13) | 0.9 |
| Multiple Sclerosis | 1.2 (0.86, 1.74) | 0.3 |
| Parkinson’s Disease | 1.2 (0.92, 1.52) | 0.2 |
| Crohn’s Disease | 0.5 (0.39, 0.68) | <0.001 |
| Muscular Dystrophy | 0.4 (0.09, 1.41) | 0.14 |
| Diverticulosis | 0.9 (0.67, 1.12) | 0.3 |
